# Survival and care practices of periviable births of <24 weeks’ gestation—a single center retrospective study in China, 2015–2021

**DOI:** 10.3389/fped.2022.993922

**Published:** 2022-12-07

**Authors:** Chun Chen, Xiaoyun Xiong, Jie Zhao, Meiqi Wang, Zhifeng Huang, Chuanzhong Yang

**Affiliations:** Department of Neonatology, Affiliated Shenzhen Maternity & Child Healthcare Hospital, Southern Medical University, Shenzhen, China

**Keywords:** survival rate, mortality, periviable infant, neonatal, extremely preterm infants, management

## Abstract

**Background:**

Data from the China Neonatal Network currently shows that the survival rate of very preterm infants in China has improved in recent years. However, due to the unequal economic and medical development of each city in China, the national data do not completely represent the level of neonatal care in the metropolitan areas. Though many studies have published their short- or long-term survival, very few have described the care practices and the course of stay of these neonates in detail. Our objective was to examine the survival and clinical practices among preterm infants born at <24 weeks’ gestational age (GA) in a high-income city in China, from 2015 to 2021.

**Methods:**

Retrospective study of preterm infants <24 weeks GA in a level 3 neonatal intensive care unit in China, over a period of 7 years (2015–2021). Care practices in neonatal intensive care units (NICU) and short- and long-term survival were measured.

**Results:**

A total of 32 periviable infants were included, with a median GA of 23.0 weeks and mean (SD) birth weight of 497 (94) g; 17 infants (53.1%) were female. While none of the infants born at 21 weeks of gestation survived until discharge, the survival rates were 25.0% (3 of 12) for infants born at 22 weeks and 58.8% (10 of 17) at 23 weeks. Antenatal corticosteroids were used in 56.3%, and 100% were vaginal birth. In the delivery room, surfactant was prescribed for 46.9% of the infants, and postnatal dexamethasone (≥2 courses) was prescribed to 61.5% of the infants. Logistic regression analysis showed that Apgar score at 5 minutes (*OR = *2.007, 95% *CI*, 1.031 to 3.906, *P < *0.05) increased the risk of death, while the increase in gestational age (*OR = *0.238, 95% *CI*, 0.060–0.936, *P < *0.05), antenatal use of steroids (*OR = *0.287, 95% *CI*, 0.106–0.778, *P < *0.01), and premature rupture of membranes (*OR = *0.141, 95% *CI*, 0.024 −0.847, *P = *0.032) could decrease the risk. No or mild neurodevelopmental impairment in surviving infants was 76.9% (10 of 13).

**Conclusions:**

Although the survival rate of periviable infants was shown to be improved in our study, there is still much room for improvement, and active follow-up information should be conducted.

## Introduction

According to the current definition of premature birth in China, babies who are born below 28 weeks of gestational age (GA) are defined as aborted babies, not infants (1). With advances in perinatal and neonatal care, more and more infants born at earlier GA had survived (2). The differences in perinatal management for infants <24 weeks result in wide variations in outcomes reported by countries. Although many studies have reported their short-or long-term outcomes, few have described in detail the nursing practice and hospitalization process of these infants. This study primarily aimed to evaluate mortality and short-and long-term outcomes among infants born <24 weeks gestation in Shenzhen, China, over the past 7 years. We present our single-center data together with our clinical practices to determine perinatal risk factors related to mortality.

## Materials and methods

### Study design and population

This retrospective cross-sectional study was carried out at the neonatal intensive care unit (NICU) of the Shenzhen Maternity & Child Healthcare Hospital. This unit has 72 incubators, serves as a level III NICU with approximately 1,500 newborn admissions per year, and is one of the largest perinatology centers in Shenzhen city, China. This is a retrospective observational study evaluating neonates of gestational ages <24 weeks born at the Shenzhen Maternity & Child Healthcare Hospital over a 7-year period from 1 January 2015–31 December 2021.Neonates who died in the delivery room were excluded from the analysis. The gestational age was determined by the best obstetric estimate based on the first obstetric ultrasound and the first day of the last normal menstrual period. Morbidity and mortality rates were retrieved from the hospital discharge records.

### Ethics

The Shenzhen Maternity and Child Healthcare Hospital Institutional Ethical Committee approved the collection and usage of the clinical information for research purposes and waived the requirement for informed consent [SFYLS No. (2019)-119].

### Data collection

All data related to the neonatal period, including sociodemographic characteristics and obstetric characteristics (such as maternal age, number of multiple births, mode of conception, and use of antenatal corticosteroids) were retrieved from the medical records of the NICU. The characteristics of neonates, i.e., sex, birth weight (BW), GA, surfactant treatment, ventilation mode, patent ductus arteriosus (PDA) and early onset neonatal sepsis, intraventricular hemorrhage (IVH), necrotizing enterocolitis (NEC), bronchopulmonary dysplasia (BPD), and retinopathy of prematurity (ROP) were recorded. All infants were examined by cranial magnetic resonance imaging (MRI) at post menstrual age (PMA) 37–40 weeks. All infants survived to discharge were followed up after discharge.

### Definitions

Antenatal steroid treatment was considered if at least one dose of dexamethasone was given 12 h before delivery. Survivors were defined as neonates who survived to the time of discharge. BPD was defined as oxygen dependency at 36 weeks of corrected age (3). ROP and the graded standard were defined by the international classification of ROP (4). IVH were diagnosed by cranial ultrasonography or MRI. The Papile grading system was used to grade IVH (5). Sepsis was defined by clinical symptoms and positive culture from blood or cerebrospinal fluid samples (6). PDA required medical or surgical treatment.

### Statistics

Continuous variables of normal distribution were expressed as the mean (±) standard deviation, and T-test was used for comparison between groups; categorical variables are expressed as number of cases and percentages. In analyses of abnormally distributed data, median (interquartile range, IQR) was used, and the Mann–Whitney *U*-test was applied for comparisons between two groups. Pearson's *χ*^2^ test or Fisher's exact test was applied in analyses of categorical variables. After adjustment for some possible confounding factors, the risk factors for mortality of periviable infants were evaluated using multiple logistic regression. In all analyses, *P* < 0.05 was taken to indicate statistical significance. The analysis was performed using SPSS statistical software version 26.0 (IBM, Corporation, NY, United States).

## Results

A total of 32 neonates born at 21^+0^–23^+6^ gestational age and admitted to NICU for active care were included in the study [21 weeks (*n* = 3), 22 weeks (*n* = 12), 23 weeks (*n* = 17)]. All infants were born by vaginal and 18 (56.3%) of the infants were exposed to antenatal corticosteroids. There was a statistically significant difference in the antenatal steroid treatment, preterm premature rupture of membrane (pPROM) rates, and Apgar score at 1 min between survivors and nonsurvivors (*P *< 0.05). The baseline characteristics of the enrolled neonates are summarized in Table 1.

**Table 1:** Demographic details of patients.

Baseline demographics	*N* = 32	Survived (*n* = 13)	Nonsurvivors (*n* = 19)	*P*-value^a^
Mean mother's age ± SD (years)	33.3 ± 5.1	32.6 ± 5.7	33.7 ± 4.7	0.567
Conception by ART, *n* (%)	12 (37.5)	7 (53.8)	5 (26.3)	0.114
Vaginal birth, *n* (%)	32 (100.0)	13 (100.0)	19 (100.0)	—
No antenatal steroid treatment	14 (43.8)	2 (15.4)	12 (66.7)	**0**.**018**
Complete dexamethasone (four doses), *n* (%)	10 (31.2)	6 (46.2)	4 (21.1)	—
pPROM, *n* (%)	13 (40.6)	8 (61.5)	5 (26.3)	**0**.**046**
Mean birth weight ± SD (g)	497.4 ± 93.8	539.8 ± 110.5	468.4 ± 69.5	**0**.**040**
Range	350–720	390–720	350–690	
Median gestational age (IQR) (weeks)	23.0 (22.3–23.6)	23.2 (22.8–23.6)	22.6 (22.2–23.2)	**0**.**036**
Range	21.4–23.6	22.2–23.6	21.4–23.6	
Female, *n* (%)	17 (53.1)	7 (53.8)	10 (52.6)	0.618
Singleton, *n* (%)	15 (46.9)	6 (46.2)	9 (47.4)	0.420
Apgar score at 1 min (IQR)	5 (2–6)	5 (5–8)	5 (1–5)	**0**.**033**
Apgar score at 5 min (IQR)	8 (5–9)	8 (6–10)	8 (5–8)	0.545
Admission temperature ± SD (°C)	35.3 ± 0.8	35.4 ± 0.7	35.3 ± 1.0	0.293
Range	(33.0–36.5)	(34.0–36.3)	(33.0–36.5)	
SGA	7 (21.9)	2 (15.4)	5 (26.3)	0.463

ART, assisted reproductive technology; pPROM, preterm premature rupture of membranes; IQR, interquartile range; SGA, small for gestational age.

^a^ Survivors vs. nonsurvivors. The statistically significant results are indicated by bold print.

While none of the infants born at 21 weeks of gestation survived until discharge, the survival rates were 25.0% for infants born at 22 weeks and 58.8% for those born at 23 weeks. A total of 13 infants survived (40.6%), and 13 of 21 (61.9%) who received active care survived. Eight of these 32 infants died on the first day, 6 died between days 1 and 7, 2 died between days 7 and 27, and 3 died between days 28 and 65. Of the infants, 15 (46.9%) used a surfactant in the delivery room. Of the survivors, two (15.4%) were classified as having severe IVH (grade 3 or 4), five (38.4%) had ROP (stage ≥3), and 10 (76.9%) had BPD. The percentages of infants who had NEC (Grade ≥2) at 22 and 23 weeks of gestation were 25.0% and 11.1%, respectively. Three infants used oxygen after discharge in total. Care practices and morbidities for the infants according to gestational age are shown in Table 2.

**Table 2:** Care practices and morbidities for the infants according to gestational age.

Variables	21 weeks (*n* = 3)	22 weeks (*n* = 12)	23 weeks (*n* = 17)	Total (*n* = 32)
Alive at discharge, *n* (%)	0 (0.0)	4 (33.3)	9 (52.9)	13 (40.6)
Day of starting feeds ± SD (h)	—	11.6 ± 1.5	10.0 ± 5.9	10.4 ± 5.2
Age when birth weight regained ± SD (days)	—	11.3 ± 2.3	11.8 ± 2.5	11.6 ± 2.3
Age when enteral nutrition ≥120 kcal/kg/d ± SD (days)	—	34.7 ± 8.4	30.2 ± 10.1	31.2 ± 9.6
Breastfeeding accounts for more than 50%^a^, *n* (%)	—	3 (75.0)	9 (100.0)	12 (92.3)
Central line duration ± SD (days)	—	136.7 ± 106.0	60.3 ± 25.8	77.9 ± 59.1
Duration of invasive mechanical ventilation ± SD (days)	—	48.3 ± 9.5	41.4 ± 13.5	43.0 ± 12.7
Duration of noninvasive mechanical ventilation ± SD (days)	—	95.3 ± 68.1	75.3 ± 54.9	79.9 ± 55.7
Total duration of supplemental oxygen ± SD (days)	—	147.3 ± 57.4	134.8 ± 55.7	137.7 ± 53.9
Length of hospital stay ±SD (days)	—	168.3 ± 86.6	152.4 ± 47.2	156.1 ± 54.5
Weight <10th percentile at discharge^a^ [*n* (%)]	—	2 (50.0)	8 (88.9)	10 (76.9)
Surfactant in the delivery room^b^, *n* (%)	1 (33.3)	7 (58.3)	7 (41.2)	15 (46.9)
Surfactant treatment^b^, ≥2 doses *n* (%)	2 (66.7)	1 (8.3)	2 (11.8)	5 (15.6)
Postnatal dexamethasone^a^, ≥2 courses *n* (%)	—	2 (50.0)	6 (66.7)	8 (61.5)
Home oxygen, *n* (%)	—	2 (50.0)	1 (11.1)	3 (23.1)
Red blood cell transfusions ± SD (times)	—	8.7 ± 2.5	5.3 ± 2.7	6.1 ± 2.9
Duration of hydrocortisone therapy ± SD (days)	—	15.0 ± 3.6	4.3 ± 6.7	6.8 ± 7.6
Range	—	(11–18	(0–20	(0–20
Duration of vasoactive drugs ± SD (days)	—	32.3 ± 21.5	15.0 ± 13.2	19.7 ± 16.7
IVH grade 3 or 4^a^, *n* (%)	—	1 (25.0)	1 (11.1)	2 (15.4)
Late-onset sepsis^a^, *n* (%)	—	2 (50.0)	1 (11.1)	3 (23.1)
ROP (stage ≥3)^a^, *n* (%)	—	2 (50.0)	3 (33.3)	5 (38.5)
BPD^a^, *n* (%)	—	4 (100.0)	6 (66.7)	10 (76.9)
NEC (Grade ≥2)^a^, *n* (%)	—	1 (25.0)	1 (11.1)	2 (15.4)
SIP^b^	—	2 (16.7)	1 (5.9)	3 (9.4)

IVH, intraventricular hemorrhage; ROP, Retinopathy of prematurity; BPD, Bronchopulmonary dysplasia; NEC, necrotizing enterocolitis; SIP, spontaneous intestinal perforation.

^a^ Data are for infants alive at discharge.

^b^ Data are for all infants.

Adjusting for other factors, the likelihood of dying in the NICU decreased significantly with increasing antenatal steroid treatment (*OR* 0.287, 95% *CI*, 0.106–0.778, *P* < 0.05). On the other hand, survival was predicted by the Apgar score after 5 min (*OR* 2.007, 95% *CI*, 1.031–3.906, *P* = 0.040) and preterm prelabor rupture of membranes (*OR* 0.141, 95% *CI*, 0.024–0.847, *P = *0.032) (Table 3).

**Table 3:** Multivariate logistic regression of independent risk factors of death.

Variables	*β*	SE	Wald	*P*	OR (95% CI)
Birth weight (g)	−0.009	0.005	3.739	0.053	0.991 (0.981–1.000)
Gestational age (weeks)	−1.435	0.699	4.219	0.040	0.238 (0.060–0.936)
Antenatal steroid treatment	−1.250	0.509	6.019	0.014	0.287 (0.106–0.778)
pPROM	−1.958	0.914	4.586	0.032	0.141 (0.024–0.847)
Apgar score at 5 min	0.697	0.340	4.202	0.040	2.007 (1.031–3.906)

pPROM, preterm prelabor rupture of membranes.

The survival rate was higher during 2020–2021 (52.6%) than during 2015–2019 (23.1%). Follow-up data with assessments at October 20, 2022, were collected for 13 infants. The proportions of motor retardation and speech delay in the evaluated infants were lower during 2020–2021 than during 2015–2019, whereas the proportion of blindness increased since one infant suffered from retinal hemorrhage (Table 4).

**Table 4:** Comparison of survival and neurodevelopmental outcomes during 2015–2019 and 2020–2021.

Variables	2015–2019	2020–2021	*P*-value
Eligible	13	19	—
Survived to hospital discharge	3/13 (23.1)	10/19 (52.6)	0.095
PMA at discharge (weeks) (mean ± SD)	45.6 ± 8.7	44.5 ± 4.9	0.833
Age^a^ at follow-up median (min–max)	5 years (3 years 9 months–6 years)	11 months (9 months–1 years 10 months)	0.011
Blindness^b^	0/3 (0.0%)	1/10 (10.0%)	0.002
Motor retardation	1/3 (33.3%)	2/10 (20.0%)	0.001
Speech delay	1/3 (33.3%)	1/10 (10.0%)	0.011

PMA, post menstrual age.

^a^ Age served as corrected age.

^b^ Blindness was defined as no functional vision in at least one eye.

## Discussion

In our single-center retrospective study, the survival rates of the periviable births <24 weeks was 40.6%, and considering active treatment was 61.9%. They are similar to that reported in Japan (NRNJ, 2013) (7); however, they were lower than the ones reported from the United States (single center, Iowa, 2020), Germany (single center, Cologne, 2016), and higher than that from France (EPIPAGE, 2015) and the United Kingdom (single center, Headington, 2021) (8–11).

Such variations may be associated with differences in willingness to provide active obstetrical and neonatal care for infants at the lowest GA. Infants that were born during the 22nd week of gestation had received resuscitation and active treatment after birth, so it is in the United States (12, 13). In China, parents were more worried about the poor outcomes of the infants and the economic burden, leading to withdrawal from treatment (14). Although our unit does selectively resuscitate according to the parents’ decision, our center's reputation of active treatment had attracted more parents who desired resuscitation and that broadened our experience in caring of these infants.

Differing clinical practices also contribute to the variability in survival rates.

Isayama (12) reported the unique aspects of management in Japan, which were different from ours. For example, most Japanese neonatologists performed functional echocardiography, applying ubiquitous sedation on all ventilated infants, monitoring the intracerebral venous velocity model in the acute phase to prevent IVH, and continuously measuring C-reactive protein (CRP) in the acute phase of infection. Watkins et al. (8) also reported other practices different from our center, such as taking high frequency oscillatory ventilation (HFOV) as the primary mode of respiratory support; few infants underwent tracheotomy. A series of measures had been taken to improve the prognosis of these infants for the past 2 years in our NICU, including delayed cord clamping, surfactant administration in the delivery room, incomplete “Golden Hour” practices in the first hour of postnatal life, and low-dose hydrocortisone supplementation within 9 days after birth, which had increased the survival rate in the past 2 years in the study. The Golden Hour protocol could significantly improve the stabilization of in extremely premature infants (15). Important elements of the “Golden Hour” in our NICU include insertion of umbilical catheters, blood sampling, surfactant administration, and the initiation of a glucose and amino acid infusion within 1 h of birth.

The lower rate of NEC in our center than others reported (8, 13) could be attributed to the nutrition management of periviable infants, that is characterized by (I) early minimal enteral feeding, (II) the promotion of breastfeeding, (III) probiotics administration, and (IV) routine glycerin enema after 7–10 days, which is appropriately similar to what Kanai et al. reported (16, 17). Mothers’ breastfeeding is actively practiced in our NICU, usually within 24–48 h after birth. Breastfed infants have significant benefits compared to formula fed infants, including reduced late infection, NEC, and improved neurodevelopmental outcomes (18). The reason for the lower rate of severe intraventricular hemorrhage in our study than the mean level in China was not clear (19). It might be due to the fact that neonatologists and NICU nurses try to minimize the handling of infants who are in the acute phase by keeping the head in the middle position, placing arterial lines for blood sampling, avoiding unnecessary oral or airway suctioning and physical examination, and unweighed within 1 week after birth in our NICU.

To our knowledge, hypothermia is a common problem, especially if delivered with less than 28 weeks gestation, which was associated with the increase of neonatal mortality and morbidities (20). The mean admission temperature of 32 periviable infants in this study was 35.3 ± 0.8°C, and the lowest was 33°C, owing to the parents withdrawing care in the delivery room due to economic burden. Our hospital is a level III maternity and child healthcare hospital. The delivery room is relatively well equipped with heat preservation equipment. Pediatricians receive each newborn in the delivery room, preheat all items in contact with the newborn in advance, and immediately place them on the incubator with the use of radiant warmers and plastic wraps to keep warm after birth, and send them to the NICU in an incubator immediately.

The main strength of our study is that it had reported in detail the various clinical practices related to the neonates born <24 weeks and discussed the differences between our practices and those reported, which has rarely been addressed before. In addition, it had tried to explore the various risk factors for mortality, and more importantly, it shows the short- and long-term outcomes of these neonates. However, this study has its limitations. It was a retrospective study and had not reported on the stillbirth rates and delivery room deaths. A relatively small sample size might result in imprecise estimates.

## Conclusions

Survival rate and clinical practices in this study differed from other centers. The data presented here provide evidence that a series of clinical improvement measures in the early postnatal life might have helped improve the survival rate of periviable infants. Although the survival rate of periviable infants was shown to be improved in this study, there is still much room for improvement.

## Data Availability

The raw data supporting the conclusions of this article will be made available by the authors, without undue reservation.
